# Drive time to cardiac rehabilitation: at what point does it affect utilization?

**DOI:** 10.1186/1476-072X-9-27

**Published:** 2010-06-04

**Authors:** Janette Brual, Shannon Gravely-Witte, Neville Suskin, Donna E Stewart, Alison Macpherson, Sherry L Grace

**Affiliations:** 1 Department of Geography, Queen's University, 99 University Avenue, Kingston, Ontario, K7L 3N6, Canada; 2 School of Kinesiology and Health Science, York University, 4700 Keele Street, Toronto, Ontario, M3J 1P3, Canada; 3 Department of Medicine - Division of Cardiology, London Health Sciences Centre and University of Western Ontario, 1151 Richmond Street, London, Ontario N6A 3K7, Canada; 4 Department of Behavioural Sciences and Health, University Health Network - Toronto General Hospital, 200 Elizabeth Street, Toronto, Ontario, M5G 2C4, Canada; 5 Department of Psychiatry, University of Toronto, 27 King's College Circle, Toronto, Ontario, M5S 1A1, Canada

## Abstract

**Background:**

A 30 minute drive time threshold has often been cited as indicative of accessible health services. Cardiac rehabilitation (CR) is a chronic disease management program designed to enhance and maintain cardiovascular health, and geographic barriers to utilization are often cited. The purpose of this study was to empirically test the drive time threshold for CR utilization.

**Methods:**

A prospective study, using a multi-level design of coronary artery disease outpatients nested within 97 cardiologists. Participants completed a baseline sociodemographic survey, and reported CR referral, enrollment and participation in a second survey 9 months later. CR utilization was verified with CR sites. Geographic information systems were used to generate drive times at 60, 80 and 100% of the speed limit to the closest CR site from participants' homes, to take into consideration various traffic conditions. Bivariate analysis was used to test for differences in CR referral, enrollment and degree of participation by drive time. Logistic regression was used to test drive time increments where significant differences were found.

**Results:**

Drive times were generated for 1209 outpatients. Overall, CR referral was verified for 523 (43.3%) outpatients, with verified enrollment for 444 (36.7%) participating in a mean of 86.4 ± 25.7% of prescribed sessions. There were significant differences in CR referral and enrollment by drive time (ps < .01), but not degree of participation. Logistic regression analysis (ps < .001) revealed that the drive time threshold at 80% of the posted speed limit for physician referral may be 60 minutes (OR = .26, 95% CI: 0.13-0.55), and the threshold for patient CR enrollment may also be 60 minutes (OR = .11, 95% CI: 0.04-0.33).

**Conclusions:**

Physicians may be taking geography into consideration when referring patients to CR. Empirical consideration also reveals that patients are significantly less likely to enroll in CR where they must drive 60 minutes or more to the closest program. Once enrolled, distance has no significant effect on degree of participation.

## Background

Cardiovascular diseases (CVD) are the leading cause of morbidity and mortality in the developed world [1]. The potential to reduce this burden of illness can be met with secondary prevention measures, such as cardiac rehabilitation (CR) [2]. Cardiac rehabilitation is a chronic disease management program designed to enhance and maintain cardiovascular health through individualized, inter-professional care. Services are provided on an outpatient basis or through home-based models of care. The benefits of CR include the management of cardiovascular risk [3], fewer hospital re-admissions [4] and a reduction in total and cardiac mortality [5]. Despite these benefits, CR remains under-utilized, with attendance rates ranging from 13 to 60% among eligible CVD outpatients [6]. Barriers of particular importance are geospatial in nature [7], such as CR site location, distribution, distance and travel time [6,8].

Longer travel times and degree of rurality have been consistently cited by patients as reasons for CR non-use [9]. However, there are few methodologically-rigorous studies about the role geographic factors play in the utilization of CR, and what evidence exists is mixed. For instance, a limited number of studies have utilized geographic information systems (GIS) technology rather than self-report indicators of CR accessibility [10-12], and even fewer studies have specifically examined these geographic factors in relation to not only referral or enrollment or participation, but to all of these phases of the utilization process [13]. No study has used GIS, in a broad sample across a wide region, to examine the relationship among geographic access and CR referral, enrollment and participation patterns.

Access to health care refers to the ability to command appropriate health care services to preserve or improve health, of which a major component is not only adequacy of supply but also physical accessibility [14]. Geographical access includes the distance and time that is required to travel to utilize health care services [15]. A 30 minute drive time standard for "accessible" hospital care was originally identified by Bosanac, Parkinson and Hall (1976) [16]. Later studies have shown that as time of travel increases over 30 minutes, patients are less likely to utilize health care services [17,18]. This drive time threshold has also been recommended by the Cardiac Care Network of Ontario (CCN) Consensus Panel in defining accessible CR [19]. However, there are no studies to our knowledge that empirically assess the applicability of a drive time threshold to secondary preventative health care such as CR. The objectives of this study were to: (1) test for significant differences in CR referral, enrollment and participation by drive time, and (2) where significant differences are found, to identify the drive time threshold affecting CR utilization.

## Methods

### Design and Procedure

**Figure 1:**
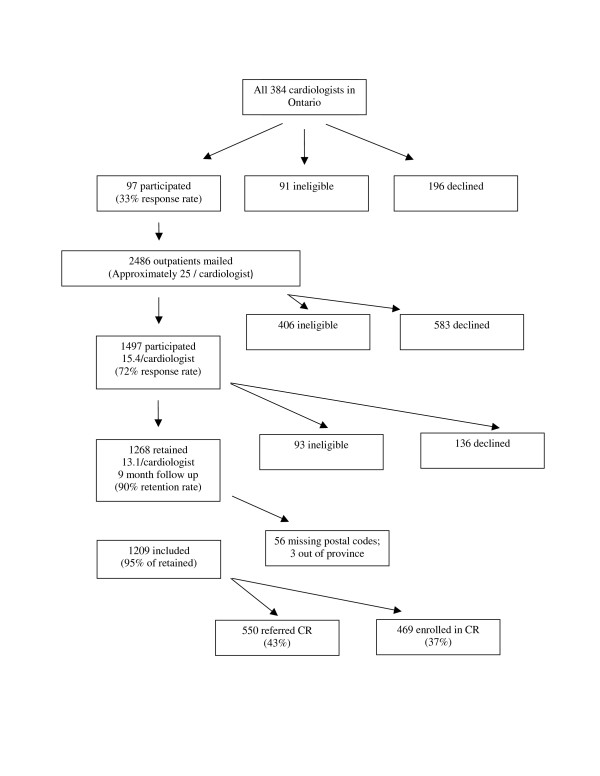
**Participant Recruitment and CR Participation**.

This study represents a secondary analysis of a larger prospective study [20]. Ethics approval was obtained from participating institutions. Three-hundred and eighty-four non-pediatric cardiologists from major centres in the Windsor to Ottawa corridor of Ontario were identified through a national physician registry, CMD Online http://www.mdselect.com, and were mailed an invitation to participate. Ninety-seven cardiologists consented to participate, and were visited by a research assistant to obtain a sample of approximately 25 each of their coronary artery disease (CAD) outpatients (see Figure 1).

With informed consent by the patients, basic clinical data was recorded from their medical charts and they were mailed a self-report survey that assessed sociodemographic characteristics among other variables. Nine months later, patients were mailed a follow-up survey that assessed their CR utilization.

A list of clinic-based CR sites in this Ontario corridor was compiled based on an exhaustive search of listings from the Canadian Association of Cardiac Rehabilitation (CACR) guidelines [21], the Cardiac Care Network of Ontario (CCN) CR pilot project report [11], the Canadian Cardiac Rehabilitation Foundation (CCRF) website http://www.cardiachealth.ca, as well as those identified by participants in the survey. Fifty-eight CR sites were identified and were each linked with patients' homes using geographic information systems (GIS) through geocoding by postal code.

### Participants

#### Cardiologists

Through the Canadian Medical Directory, all 384 cardiologists with practices in a "major market" from the Windsor to Ottawa corridor of Ontario, including Windsor, London, Kitchener-Waterloo-Cambridge, Hamilton/Burlington, Metro Toronto, Kingston, and Ottawa were identified and invited to participate. Physician inclusion criterion was having an active, non-pediatric practice. Ninety-seven cardiologists consented to participate (33% response rate; see Figure 1). Of the 384 cardiologists approached, 91 were considered ineligible for the following reasons: non-CAD patients (n = 57; 62.6%), no outpatient practice (n = 12; 13.2%), incorrect physician address/no longer in practice (n = 9; 9.9%), retired from clinical practice (n = 2; 2.2%) or other reasons such as physician on sabbatical or maternity leave, left the country, illness, or had an independent practice not covered under hospital ethics approval (n = 11; 12.1%). Of the consenting cardiologists, a sample of approximately 25 of their CAD outpatients was identified for potential participation in the study.

#### Patients

CAD outpatients were eligible to participate in the study, but specifically confirmed acute coronary syndrome (ACS) patients were targeted or those having recently undergone coronary artery bypass grafting or percutaneous coronary intervention. Diagnosed CAD was confirmed based on the medical history indicated in the patient's chart, through documented physical examination, diagnostic ECG changes (i.e., Q waves, and/or ST-T segment changes), and/or troponin levels above the 99^th ^percentile of normal.

Patient inclusion criteria were: eligibility for CR based on CACR guidelines [21] and English language proficiency. Two thousand four-hundred and eighty-six outpatients were invited to participate, and 1497 consented at baseline, representing a mean of 15.4 patients per cardiologist (406 ineligible, 72% response rate; see Figure 1). Based on the larger study, reasons for ineligibility included lack of English language proficiency (n = 145; 35.7%), could not locate the patient (n = 86; 21.2%), other reasons such as moved out of the country (n = 39; 9.6%), no CAD diagnosis (n = 37; 9.1%), deceased (n = 34; 8.4%), orthopedic, neuromuscular, cognitive or vision impairment (n = 33; 8.1%), outdated index event or treatment (n = 18; 4.4%), ineligibility for CR based on Canadian guidelines [21] (n = 6; 1.5%), previous attendance at CR (n = 5; 1.2%), and non-dysphoric or anxious psychiatric conditions (n = 3; 0.7%).

At the nine-month follow up, 1268 patients returned their mailed survey (93 ineligible at follow-up; 90% retention rate; see Figure 1). Reasons for ineligibility for follow-up included inability to locate the patient (n = 37; 41.1%), deceased (n = 23; 25.6%), other reasons such as too ill to participate or moved out of the province/country (n = 27; 26.6%), and new onset orthopedic, neuromuscular, cognitive or vision impairment or non-dysphoric or anxious psychiatric condition (n = 6; 6.7%). This represents a mean of 13.1 patients per cardiologist. For the geospatial analysis of CR utilization, 1209 patients were included. Distances and drive times were not generated for 56 (4.4%) outpatients due to missing or invalid postal codes and three (0.2%) patients who lived out of province.

### Measures

At baseline, sociodemographic variables assessed in the self-report survey included marital status, ethnocultural background, gross annual family income, level of education attained and work status through forced-choice response options. Age, sex and clinical data including previous cardiac events, disease severity and risk factors were extracted from outpatient charts.

The Duke Activity Status Index (DASI; [22]) was administered as an indicator of functional capacity. The DASI is a brief 12-item, self-administered survey. Participants were questioned about their ability to perform common activities of daily living, such as personal care, ambulation, household tasks, sexual function, and recreational activities, which are each associated with specific metabolic equivalents (METs). This is a valid and commonly used tool, which correlates highly with peak oxygen uptake [23]. Higher scores on the DASI correspond with a greater functional capacity.

### Spatial Analysis and Geographic Variables

A customized application developed by Healthcor Inc. (Toronto, Ontario) was used to generate distances in kilometres and drive times in minutes. The core geographical information system software used was Microsoft MapPoint 2006 [24]. Outpatients' home postal codes and all 58 CR sites' postal codes were geocoded to create multiple start and destination points. By linking the outpatients' home postal codes to each of the CR site postal codes start-to-destination pairs were generated and the shortest distances and drive times were derived using the Ontario Road Network (ORN) file. The ORN is a detailed geospatial database of the province of Ontario's road networks that consists of interconnected vectors and includes information such as road length, class (e.g., highways, streets) and direction (i.e., one-way or two-way) [25].

The application was used to determine the shortest route to travel, correcting for one-way streets and used maximum road speeds based on the class of the road (e.g., 100 kilometre/hour for highways). Three drive times were generated using percentage adjustments, and were classified as no traffic, non-rush hour and rush hour drive times. The purpose of the percent adjustments was to reflect actual average speeds using the posted road speed maximums as the base, which would change during rush hours to consider stop signs, traffic signals and congestion. No traffic drive times reflect travel at 100% of the posted speed limit, non-rush hour drive times reflect travel at 80% of the speed limit, and rush hour drive times reflecting travel at 60% of the speed limit. This method of percent adjustments was utilized to quantitatively standardize road and traffic features for all start-to-destination routes calculated and was developed by Healthcor Inc. Independent studies on CR utilization in Ontario, Canada, as well as other businesses and retailers have consulted previously with Healthcor Inc., utilizing similar drive time analyses [11,19].

### Cardiac Rehabilitation Utilization

In the 9-month follow-up survey, respondents were asked whether or not they were referred to, and enrolled in (i.e., attendance at an intake appointment) CR (yes/no). This included referral by their cardiologist or other healthcare providers. Outpatients reporting that they attended CR were asked to report the percentage of prescribed sessions they attended. CR utilization was verified with 41 sites to which patients reported referral.

### Statistical Analyses

Once cleaned and screened, a descriptive analysis of the data was performed using SPSS 15.0 [26] to summarize the sociodemographic characteristics of the study population. Differences in retained, ineligible and declining patients at the 9 month follow-up assessment were compared using ANOVAs and chi-square analyses as appropriate. With regard to the former, where significant Post-hoc Bonferroni tests were used to test differences by participant status.

A descriptive examination of CR utilization was performed for 1209 (95%) outpatients. T-tests were used to investigate differences in CR referral and enrollment by drive time to the closest CR program. Pearson's correlation was used to examine the relationship between distance and percentage of sessions attended among enrollees. Finally, CR drive times were categorized into 10 minute increments from 0 through to over 80 minutes. In order to ensure adequate numbers in each increment, those who drove between 60 and 80 minutes were grouped together as were those who drove more than 80 minutes. Logistic regression analyses were used to examine the relationship between these drive time increments and (1) CR referral and (2) enrollment.

## Results

### Respondent Characteristics

**Figure 2:**
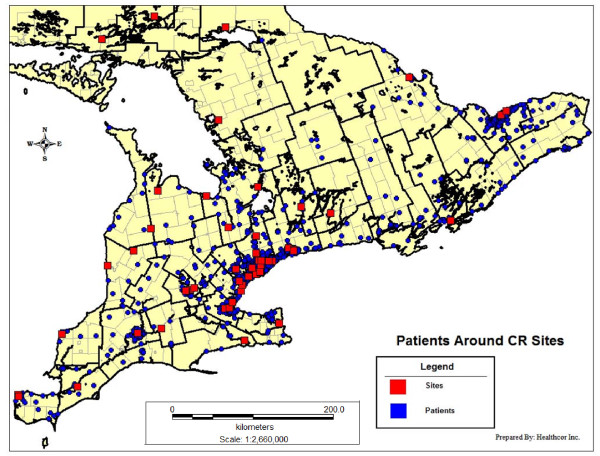
**Spatial distribution of participants and CR sites in the Southern corridor of Ontario**.

Figure 2 maps the distribution of participants' residences and CR sites within the study area of the Southern corridor of Ontario. Characteristics of retained, ineligible and declining patients at the 9 month follow up assessment are shown in Table 1. Overall differences are denoted in the total column, with specific differences from retained participants denoted in the other columns. There were some significant differences, with retained participants more likely to be older, married or partnered, white, to have a family income greater than $50,000CAD, lower diastolic BP and higher functional status than non-participants.

**Table 1:** Characteristics of participating, ineligible and declining outpatients at 9 month follow-up

Characteristic	Retained Participants (n = 1268)	Ineligibles (n = 93)	Declined (n = 136)	Total (N = 1497)
**Sociodemographic**				
Age (mean ± SD)	66.3 ± 11.2	65.4 ± 13.4	63.6 ± 12.2*	65.9 ± 11.4*
Sex (% female)	358 (28.2)	29 (31.2)	43 (31.6)	430 (28.7)
Living status† (%alone)	289 (23.0)	29 (31.2)	35 (26.1)	353 (23.8)
Marital status† (%married/partnered)	910 (72.3)	57 (61.3)*	81(60.0)*	1048 (70.5)**
Ethnocultural background† (% white)	1094 (86.3)	72 (77.4)*	101 (74.3)***	1267 (84.6)***
Family income† (% ≥ $50,000CAD)	560 (48.5)	28 (31.8)**	43 (37.4)	631 (46.5)**
Education† (% >high school)	670 (53.7)	42 (45.7)	70 (52.2)	782 (53.1)
Work status† (% fulltime/part-time)	406 (32.3)	28 (30.4)	52 (39.6)	487 (32.8)
**Clinical**				
BMI† (mean ± SD)	27.5 ± 5.4	27.7 ± 5.9	27.6 ± 5.7	27.5 ± 5.4
Systolic BP mm Hg (mean ± SD)	131.2 ± 19.1	135.0 ± 21.1	130.3 ± 19.8	131.3 ± 19.3
Diastolic BP mm Hg (mean ± SD)	74.5 ± 10.2	77.5 ± 112.9*	73.3 ± 10.4	74.6 ± 10.5*
Total Cholesterol/HDL Ratio (mean ± SD)	4.2 ± 1.2	4.1 ± 1.1	4.1 ± 1.2	4.2 ± 1.2
HDL mmol/L (mean ± SD)	1.2 ± 0.4	1.1 ± 0.3	1.2 ± 0.3	1.2 ± 0.4
LDL mmol/L (mean ± SD)	2.3 ± 0.9	2.1 ± 1.0	2.5 ± 0.9	2.3 ± 0.9
NYHA Class II-IV (%)	82 (48.5)	5 (41.7)	6 (31.6)	93 (6.2)
CCS angina class 2-4 (%)	262 (81.9.7)	7 (58.3)	25 (71.4)	294 (80.1)
Multi-vessel Disease (>1 diseased coronary arteries)	365 (79.5)	25 (83.3)	34 (82.9)	424 (80.0)
Duke Activity Status Index† (mean ± SD)	37.2 ± 15.8	28.0 ± 18.9***	34.1 ± 16.2*	36.4 ± 16.2***
Current or Previous MI	578 (88.7)	46 (90.2)	65 (87.8)	689 (88.7)
Current or Previous PCI	558 (90.3)	38 (88.4)	54 (94.7)	650 (90.5)
Current or Previous CABG	360 (80.7)	19 (70.4)	31(81.6)	410 (80.2.4)
Current or Previous HF	177 (75.0)	20 (87.0)	24 (80.0)	221 (76.5)
Current or Previous of Valve repair/replacement	194 (85.5)	15 (78.9)	27 (81.8)	236 (84.6)
**Geographic:**				
Distance km (mean ± SD)	17.4 ± 23.1	15.5 ± 20.4	14.4 ± 18.8	17.0 ± 22.6
No Traffic Drive Time, min (mean ± SD)	21.9 ± 21.9	19.7 ± 19.1	19.3 ± 16.6	21.5 ± 21.4
Non-rush hour Drive Time, min (mean ± SD)	27.4 ± 27.5	24.6 ± 23.9	24.2 ± 20.8	26.9 ± 26.7
Rush hour Drive time, min (mean ± SD)	36.5 ± 36.6	32.8 ± 31.8	32.2 ± 27.7	35.9 ± 35.6

† denotes data from patient report. All other data elements extracted from patient charts.

*p < 0.05, **p < 0.01, ***p ≤ 0.001

Note: Percentages take into account missing data for some variables.

SD, standard deviation; BMI, Body Mass Index; BP, blood pressure; HDL, high-density lipoprotein; LDL, low-density lipoprotein CCS, Canadian Cardiovascular Society; NYHA, New York Heart Association. MI, Myocardial Infarction; PCI, Percutaneous Coronary Intervention; CABG, Coronary Artery Bypass Grafting; HF, Heart Failure; Min = minutes; Km = kilometers

### Cardiac Rehabilitation Distance and Drive Times

As assessed through objective geographic means, the mean distances and drive times to the closest CR site for the total sample are shown in Table 1. The Ontario sample had a median distance of 7.3 km (range, 0.5-176.76) to the closest CR site and a median non-rush hour drive time of 16.6 min (range, 0-252.97).

### Cardiac Rehabilitation Utilization

Overall, CR site-verification of self-reported utilization with 41 programs revealed that 523 (43.3%) outpatients were referred to CR and 444 (36.7%) enrolled. Over 85% of referred outpatients enrolled in CR. Enrolled outpatients attended a mean of 86.4 ± 22.6% of prescribed and site-verified CR sessions.

### Cardiac Rehabilitation Utilization by Drive Time

Overall, the median distance for outpatients who were referred to CR was 6.9 km (range, 0.0-118.5) compared to 7.8 km (range, 0.0-176.8) for those who were not. The median non-rush hour drive time for referred outpatients was 16.1 min (range, 0.0-159.3) compared to 17.2 min (range, 0.5-253.0) for those not referred. CR enrollees had a median distance of 6.4 km (range, 0.0-97.5) and median non-rush hour drive time of 15.8 min (range, 0.0-128.1), compared to a median distance of 8.1 km (range, 0.0-176.8) and median non-rush hour drive time of 17.4 min (range, 0.0-252.9) for non-enrollees.

As shown in Table 2, both referral and enrollment were significantly related to drive time to the closest CR program from a patient's home, with significantly greater drive times leading to non-referral and non-enrollment. Degree of CR participation among enrollees was not significantly related to drive time (r = -.02, p = .63).

**Table 2:** CR-site verified referral and enrollment by drive time to the closest program

	N (%)	†Drive Time (mean ± SD)	P
Referral			
Yes	523 (43.3%)	23.26 ± 21.36	< .001
No	686 (56.7%)	30.50 ± 30.96	

Enrollment (of all, N = 1268)			
Yes	444 (36.7%)	20.93 ± 17.63	< .001
No	765 (63.3%)	31.12 ± 31.20	

Enrollment (of those referred, n = 523)			
Yes	444 (84.9%)	20.93 ± 17.63	< .001
No	79 (15.1%)	36.37 ± 32.92	

TOTAL	1209 (100%)	27.4 ± 27.45	

† Non-rush hour drive time in minutes

Results of logistic regression analyses examining 10 minute increments in drive times and their relationship to each of CR referral and enrollment are summarized in Tables 3, 4 and 5. The tables display the results for 60%, 80% and 100% of the maximum posted speed limit, to reflect CR utilization under different traffic and road conditions. The models adjusted for age, sex and activity status at baseline.

**Table 3:** Relationship of rush hour drive time increments to verified referral and enrollment in CR, N = 1209

Rush Hour Drive Time - 60%		Model 1: CR Referral		Model 2: CR Enrollment	
	**n (%)** **overall**	**n (%)** **referred**	**OR (95% CI)**	**n (%)** **enrolled**	**OR (95% CI)**

Sex (male)			.92 (.70-1.20)		.98 (.75-1.31)
Age			.98 (.97-1.00)**		.99 (.98-1.00)*
Baseline Activity Status			1.01 (1.01-1.02)**		1.02 (1.01-1.02)***
Drive Time					
Less than 10 minutes	141 (11.7%)	59 (11.3%)	Reference	52 (11.7%)	reference
10 - 20 minutes	365 (30.2%)	167 (31.9%)	1.24 (.83-1.85)	149 (33.6%)	1.24 (.82-1.86)
20 - 30 minutes	272 (22.5%)	136 (26.0%)	1.38 (.91-2.09)	123 (27.7%)	1.39 (.91-2.12)
30 - 40 minutes	101 (8.5%)	48 (9.2%)	1.17 (.69-1.98)	40 (9.0%)	1.03 (.61-1.76)
40 - 50 minutes	66 (5.5%)	28 (5.4%)	.98 (.54-1.78)	22 (5.0%)	.82 (.44-1.53)
50 - 60 minutes	48 (4.0%)	20 (3.8%)	1.01 (.51-1.98)	17 (3.8%)	.95 (.48-1.91)
60 - 80 minutes	81 (6.7%)	34 (6.5%)	.96 (.54-1.69)	25 (5.6%)	.72 (.40-.1.31)
Over 80 minutes	135 (11.2%)	31 (5.9%)	.40 (.24-.68)**	16 (3.6%)	.22 (.11-.42)***
TOTAL	1209 (100%)	523 (100%)		444 (100%)	
Note: 56 outpatients had missing drive times and 3 lived out of province and were therefore excluded from analysis.					
OR = Odds Ratio CI = Confidence Interval *p < 0.05, **p < 0.01, ***p ≤ 0.001					

Note: 56 outpatients had missing drive times and 3 lived out of province and were therefore excluded from analysis.

OR = Odds Ratio

CI = Confidence Interval

*p < 0.05, **p < 0.01, ***p ≤ 0.001

**Table 4:** Relationship of non-rush hour drive time increments to verified referral and enrollment in CR, N = 1209

Non-Rush Hour Drive Time- 80%		Model 1: CR Referral		Model 2: CR Enrollment	
	**n (%)** **overall**	**n (%)** **referred**	**OR (95% CI)**	**n (%)** **enrolled**	**OR (95% CI)**

Sex (male)			.92 (.70-1.20)		.98 (.75-1.30)
Age			.98 (.97-.99)**		.99 (.97-1.00)*
Baseline Activity Status			1.01 (1.01-1.02)**		1.02 (1.01-1.02)**
Drive Time					
Less than 10 minutes	257 (21.3%)	120 (22.9%)	reference	106 (23.9%)	reference
10 - 20 minutes	463 (38.3%)	210 (40.2%)	.97 (.71-1.32)	189 (42.6%)	.99 (.73-1.37)
20 - 30 minutes	159 (13.2%)	80 (15.3%)	1.03 (.69-1.54)	69 (15.5%)	.97 (.65-1.46)
30 - 40 minutes	79 (6.5%)	35 (6.7%)	.86 (.51-1.44)	28 (6.3%)	.74 (.44-1.26)
40 - 50 minutes	61 (5.0%)	24 (4.6%)	.67 (.38-1.21)	20 (4.5%)	.63 (.34-1.15)
50 - 60 minutes	55 (4.5%)	23 (4.4%)	.76 (.41-1.38)	16 (3.6%)	.54 (.28-1.02)
60 - 80 minutes	36 (3.0%)	10 (1.9%)	.26 (.13-.55)***	4 (.9%)	.11 (.04-.33)***
Over 80 minutes	84 (6.9%)	21 (4.0%)	.35 (.20-.62)***	12 (2.7%)	.22 (.11-.43)***
TOTAL	1209 (100%)	523 (100%)		444 (100%)	

Note: 56 outpatients had missing drive times and 3 lived out of province and were therefore excluded from analysis.

OR = Odds Ratio

CI = Confidence Interval

*p < 0.05, **p < 0.01, ***p ≤ 0.001

**Table 5:** Relationship of no traffic drive time increments to verified referral and enrollment in CR, N = 1209

Drive Time - 100%		Model 1: CR Referral		Model 2: CR Enrollment	
	**n (%)** **overall**	**n (%)** **referred**	**OR (95% CI)**	**n (%)** **enrolled**	**OR (95% CI)**

Sex (male)			.93 (.71-1.21)		1.00 (.76-1.32)
Age			.98 (.97-1.00)**		.99 (.98-1.00)*
Baseline Activity Status			1.01 (1.01-1.02)**		1.02 (1.01-1.02)***
Drive Time					
Less than 10 minutes	392 (32.4%)	178 (34.0%)	Reference	159 (35.8%)	Reference
10 - 20 minutes	429 (35.5%)	204 (39.0%)	1.05 (.79-1.38)	182 (41.0%)	1.03 (.77-1.37)
20 - 30 minutes	124 (10.3%)	56 (10.7%)	.87 (.58-1.32)	45 (10.1%)	.74 (.48-1.13)
30 - 40 minutes	74 (6.1%)	31 (5.9%)	.83 (.50-1.39)	26 (5.9%)	.76 (.45-1.29)
40 - 50 minutes	66 (5.5%)	25 (4.8%)	.67 (.39-1.15)	17 (3.8%)	.46 (.26-.84)*
50 - 60 minutes	33 (2.7%)	6 (1.1%)	.27 (.11-.67)**	3 (0.7%)	.15 (.04-.49)**
60 - 80 minutes	48 (4.0%)	15 (2.9%)	.49 (.26-.95)*	9 (2.0%)	.31 (.14-.66)**
Over 80 minutes	43 (3.6%)	8 (1.5%)	.25 (.11-.55)**	3 (0.7%)	.10 (.03-.33)***

TOTAL	1209 (100%)	523 (100%)		444 (100%)	

Note: 56 outpatients had missing drive times and 3 lived out of province and were therefore excluded from analysis.

OR = Odds Ratio

CI = Confidence Interval

*p < 0.05, **p < 0.01, ***p ≤ 0.001

With regard to referral, the overall models were significant for all 3 drive times (ps < 0.001). For rush hour drive times or 60% of the maximum speed limit, the drive time threshold for physician referral was 80 minutes. A 60 minute non-rush hour (80% of the maximum speed limit) drive time to the closest CR program was the threshold significantly related to CR referral. For no traffic drive times (100% of the maximum speed limit), physicians were likely to refer to outpatients to CR who lived within 50 minutes. Overall, 1,074 (88.8%) outpatients lived within a 60 minute drive time of a CR program based on non-rush hour conditions.

With regard to enrollment, the overall models were significant for all 3 drive times (ps < 0.001). An 80 minute rush hour drive time to closest CR was the threshold significantly related to CR enrollment. For non-rush hour drive times to the closest CR program the threshold related to CR enrollment was 60 minutes. For no traffic drive times referred patients were likely to enroll in CR if they lived within 40 minutes of a program.

## Discussion

To the best of our knowledge, this is the first study to utilize GIS to objectively assess the relationships between drive time and CR referral, enrollment and participation within a broad sample of cardiac outpatients. There was a range in drive time to the closest CR program between 0 and 252.9 minutes, with a median of 16.6 minutes. Overall results revealed a significant relationship between geographic access and CR referral and enrollment, but not degree of participation once enrolled. Specifically, when assuming a driving speed of 80% of the posted limit, outpatients were significantly less likely to be referred and enroll with drive times over 60 minutes.

In the overall literature, few studies have examined CR utilization in samples living in regions of various population densities [9,13,27-30]. There are a limited number of studies that have utilized GIS technology rather than self-report indicators of CR accessibility [10-12], and at the time of this report only one study that had examined these geographic factors in relation to not only referral or enrollment or participation, but to all of these phases of the utilization process [13]. No study has used GIS, in a broad sample across a wide region, to examine the relationship among geographic access and CR referral, enrollment and participation patterns.

The overall finding of a relationship between CR utilization and geographic access is congruent with eight studies in this literature [9-11,27,29-32], but discordant with four studies [12,13,28,33]. Of these latter studies, three [13,28,33] utilized self-reported measures to assess geographic access, which can result in an over- or underestimation of distance-related barriers. For example, the study by Melville, Packham, Brown, Weston and Gray (1999) consisted of two cohorts of 878 patients who were admitted to hospital with myocardial infarction who lived within 0-24 km of the CR site [12]. Due to this restricted range, these results may underestimate the role of distance as a barrier to CR, given that all participants lived within the 20 mile or 32 km threshold of "accessible" healthcare services [16]. Therefore, the overall literature does support the important role of geospatial access in CR referral and enrollment.

The results from this study compare access to CR when taking into consideration actual traffic constraints that patients generally face. As expected, drive time thresholds for referral and enrollment were greatest when assuming high traffic congestion (60% of maximum posted speed; 80 minutes), and lowest assuming no traffic conditions (100%; 50 minutes for referral and 40 minutes for enrollment). The referral and enrollment drive time thresholds were the same at both rush hour and non-rush hour drive times, however they were 10 minutes less for enrollment for the no-traffic drive time (50 versus 40 minutes). While overall we conclude based on the results that an appropriate drive time threshold for CR be 60 minutes, if a particular program is situated in a high-traffic area, a more conservative threshold should potentially be considered. It may be warranted to speak individually to patients referred to these CR programs and consider triaging to home-based models where patients will be accessing services during rush hour and will have a greater than 40 minute drive time.

Assuming a non-rush hour context, the threshold for both physician referral and outpatient enrollment were both 60 minute drive times, which suggests that physician referral practices may not influence uptake to CR. However, where a CR site is not available in a reasonable distance or drive time to a patient's home, referral to a home-based CR program would be indicated. Home-based CR is shown to have equivalent benefits to hospital-based CR [34]. Previous research by our group has shown that referral to home-based CR services is not more likely in the presence of geographic barriers [35]. Increasing physician awareness of the availability and efficacy of such services may aid in increasing CR referrals and patient enrollment in the presence of geographic barriers.

While CR enrollment varied by drive time, there were no significant differences in degree of CR participation by drive time. Results suggest that if patients are willing to enroll at distances less than 60 minutes, then within that threshold these drive times do not seem to affect session adherence. This confirms the lack of association reported between geospatial indicators and degree of participation in a rural American sample [33]. These results also suggest that once patients access CR services, they are highly satisfied with the program.

Almost three-quarters of the overall sample could attend a CR program within a 30 minute drive time, considered 'accessible' in the primary healthcare and policy literature, and by the Cardiac Care Network of Ontario Consensus Panel [19]. These results are comparable to the Ontario CR Pilot Project which found that 66% of patients could access CR within 30 minutes [11]. However, there is no empirical research to test what an appropriate threshold for accessibility might be for outpatient services. Attending CR generally involves a commitment of two on-site visits per week for approximately six months. By examining 10 minute increments in drive time to the closest CR program, results showed that physicians are less likely to refer patients who have a greater than 60 minute drive to access CR. Results also showed that patients are significantly less likely to enroll with drive times greater than 60 minutes. Over 94% of the referred sample could access CR within 60 minutes. Given this is the first empirical test of a drive time cut-off for CR enrollment, clearly replication is warranted before policy transfer can be undertaken.

CR siting decisions in the province have not been made with an eye to regional need, but have rather arisen through local initiative. An examination of the distribution of CR programs, capacity by need, and identification of gaps was conducted through the provincial CR Pilot Project [11]. Conclusions pointed to room for improvement in siting programs to optimize patient access. However, the policy question arises as to what density of patients is required to warrant the cost and resources necessary to run another CR program? At what point would it be acceptable to offer home-based CR services (which would ensure equitable access to health care services for rural inhabitants) rather than establish another program? The results herein suggest that most patients in the relatively densely populated region of Southern Ontario have adequate access to CR with regard to drive times. This study however cannot speak to whether the programs as sited have sufficient capacity to provide services to patients within a 60 minute catchment area.

Caution is warranted when interpreting these results. First, we did not capture information regarding the mode of transportation that outpatients used to travel to CR such as public transportation, taxi, or carpool for example, but assumed travel by car for all participants. Parking costs and availability, or poor driving conditions may indirectly pose as transportation barriers but were not specifically addressed in the study. Secondly, the drive times generated were based on the assumption that outpatients were referred to the closest CR site. Due to physician or patient preferences or wait lists, patients may not have attended the closest CR site, and indeed this will form the basis for future study. Third, study results may be limited in generalizability. The sample was recruited in the densely populated region of Southern Ontario, with a small rural sample relative to the overall study. Moreover, results may not be generalizable to other health care systems, particularly those where CR services are not covered. Finally, study participants were more likely to be older, married or partnered, white, to have a family income greater than $50,000CAD, lower diastolic BP and higher functional status than non-participants. This suggests that the results herein may be more applicable to cardiac outpatients with higher socioeconomic status who are in relatively good health. Replication is warranted in other settings before these results are used in policy decisions.

## Conclusions

Overall, the results from this study suggest that CR utilization varied significantly by drive time, with outpatients at a greater geographic disadvantage being significantly less likely to be referred and enroll in CR. Based on empirical considerations, our data suggest that CR enrollment varies with traffic conditions. During rush hour conditions, enrollment was unaffected with drive times of less than 80 minutes. CR enrollment was unaffected with non-rush hour drive times of less than 60 minutes, while no traffic drive times below 40 minutes did not influence enrollment. In the presence of geographic barriers, referral to home-based services should be considered. Over 88% of this broad outpatient sample lived within 60 minutes of a CR program in non-rush hour conditions compared to just over 82% during rush hour and over 92% for those in no traffic, suggesting adequate access to CR. Once enrolled, drive times were unrelated to degree of CR participation.

## Competing interests

The authors declare that they have no competing interests.

## Authors' contributions

All of the above named authors have contributed sufficiently to this manuscript. JB acquired data, analyzed and interpreted the data and drafted the manuscript. SGW participated in the study coordination, acquired data and helped to draft the manuscript. NS, DES and AM revised the manuscript critically for important intellectual content and contributed to interpretation of the data. SLG conceived of the study and its design, and aided in drafting the manuscript and revised it critically for important intellectual content. All authors read and approved the final manuscript.
